# A Distance-Adaptive Method for Three-Axis Angle Measurement Based on an Optical Wedge

**DOI:** 10.3390/s26144430

**Published:** 2026-07-12

**Authors:** Jinkai Wang, Chengping Ran, Lihui Wang, Lizhong Zhang, Yangyang Bai

**Affiliations:** 1 College of Mechanical and Electrical Engineering, Changchun University of Science and Technology, Changchun 130022, China; 2 College of Optoelectronic Engineering, Changchun University of Science and Technology, Changchun 130022, China; 3 College of Engineering, Inner Mongolia Minzu University, Tongliao 028000, China; 4 Key Laboratory of Air-to-Ground Laser Communication for National Defense, Changchun University of Science and Technology, Changchun 130022, China

**Keywords:** three-axis angle measurement, optical wedge, distance-adaptive method, autocollimation, error compensation

## Abstract

Existing non-contact three-axis angle-measurement methods are unsuitable for measuring the relative three-axis angles between the inner and outer ring frames of fifth-generation airborne optoelectronic gimbal platforms. Our previous study proposed a compact three-axis angle-measurement method based on an optical wedge for this application. However, the fixed-coefficient angle-solving model used in that method does not account for variations in the measurement distance *L*, which can produce distance-dependent nonlinear errors when axial displacement of the inner ring frame occurs. To address this limitation, the present study proposes a distance-adaptive method for three-axis angle measurements. An analytical measurement-distance model is established using ABCD ray-transfer-matrix theory, and *L* is incorporated into the fixed-coefficient angle-solving model. The fixed coefficients in the polynomial error-compensation model are thereby expressed as functions of *L* and updated according to the estimated measurement distance. Experimental results demonstrate that the proposed method significantly improves the measurement accuracy under varying-distance conditions. Taking the measurement-distance condition with the largest error, *L* = −2 mm, as an example, the RMS values of the measurement errors for the pitch, yaw, and roll angles are reduced from 13.0″, 8.4″, and 31.1″ to 5.6″, 3.8″, and 18.6″, respectively.

## 1. Introduction

With the increasing requirements for line-of-sight stability in airborne reconnaissance, precision guidance, and high-resolution imaging missions, the accuracy of attitude sensing and closed-loop control in airborne optoelectronic gimbal platforms has become an important factor limiting system performance [1,2]. For fifth-generation airborne optoelectronic gimbal platforms without a physical inner ring frame mechanism, real-time feedback of the relative three-axis angles between the inner and outer ring frames directly determines the line-of-sight pointing accuracy and the control performance of the servo system [3]. Therefore, achieving high-precision, non-contact, real-time measurements of the three-axis angles between the inner and outer ring frames within the space-constrained region has become a critical issue to be addressed. Existing sensors, such as rotary transformers, optical encoders, and capacitive angular position sensors, struggle to meet the requirements for accurate three-axis angle measurement under the coupled translational and rotational motions of the two frames. Meanwhile, owing to the restricted measurement space between them, existing non-contact three-axis angle measurement methods cannot be integrated because of their large size and layout constraints [4,5,6,7,8,9]. In addition, the target platform requires RMS measurement errors of less than 0.01° for pitch and yaw and less than 0.1° for roll, together with a measurement update rate of at least 1000 Hz.

To address these limitations, our previous study proposed a lightweight and compact three-axis angle-measurement method based on an optical wedge [10], and a fixed-coefficient angle-solving model was established based on the mapping relationship between the centroid coordinates of the two spots and the three-axis angles. By utilizing the two reflection paths formed by the front and rear surfaces of the optical wedge, this method can simultaneously obtain the three-axis angle information of the measured target without requiring multiple beam deflections using beam splitters or plane mirrors. The method offers the advantages of a simple structure, fast response, and a small measurement space requirement. The feasibility of the proposed measurement method and the validity of the fixed-coefficient angle-solving model were experimentally verified. The basic optical configuration, the initial angle-solving equations, the fixed-coefficient polynomial error-compensation model, and the experimental platform are retained from Ref. [10] as the baseline of the present work. Building on this baseline, the present study focuses on improving three-axis angle-measurement accuracy under varying measurement-distance conditions through the proposed analytical measurement-distance model and distance–angle nested model.

The aforementioned fixed-coefficient angle-solving model was established without considering variations in measurement distance. In practical engineering applications, because the fifth-generation airborne optoelectronic gimbal platform employs a parallel flexible support structure, the inner ring frame inevitably undergoes slight axial translation along the optical axis of the measurement system when moving relative to the outer ring frame, thereby causing variations in the measurement distance. According to the engineering-level specification of the target pre-research project on a fifth-generation airborne optoelectronic gimbal platform, the allowable axial relative displacement between the inner and outer ring frames is ±2 mm. With a nominal measurement distance of 10 mm, this requirement corresponds to a physical working-distance range of 8–12 mm. Therefore, the distance range considered in this study represents the project-specific engineering operating range of the target prototype.

According to the principle of autocollimation imaging, these variations alter the image-side magnification of the system. Even when the attitude of the optical wedge remains unchanged, the spot centroids on the image plane shift slightly. For short-focal-length autocollimation systems, even a minor shift in the centroid of the light spot can result in significant measurement errors, causing the angle measurement results based on the fixed-coefficient angle-solving model to exhibit nonlinear errors related to the measurement distance. To address this issue, the pitch-angle calculation for *α* was modified in our previous study by exploiting the opposite *y*-direction displacements of the primary- and secondary-path spots induced by measurement-distance variations. Specifically, the mean of the *y*-coordinates of the two spot centroids was used in place of the centroid coordinate of a single spot in the pitch-angle calculation, thereby partially suppressing the influence of measurement-distance variation. However, because the measurement distance is not included as an independent variable in the fixed-coefficient angle-solving model, the method remains essentially empirical. When the distance varies, the model coefficients cannot be adaptively updated, leading to reduced angle measurement accuracy and degraded measurement consistency.

To suppress the measurement errors induced by axial displacement of the inner ring frame, a distance-adaptive method for three-axis angle measurements is proposed to reduce the sensitivity of the angle measurement results to distance. First, an analytical measurement-distance model is established based on ABCD ray-transfer-matrix theory [11], and *L* is introduced as an explicit variable into the fixed-coefficient angle-solving model. The originally fixed compensation coefficients are expressed as functions of *L*, thereby establishing the distance–angle nested model. By determining *L* in real time and updating the coefficients of the nested model, the proposed method achieves high-precision three-axis angle measurements under varying-distance conditions.

## 2. Principle and Modeling of the Distance-Adaptive Method for Three-Axis Angle Measurement

### 2.1. Principle of Three-Axis Angle Measurement Based on an Optical Wedge

**Figure 1:**
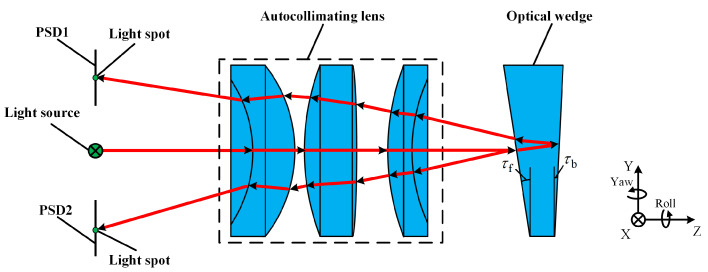
Schematic diagram of beam propagation path.

The compact three-axis angle measurement system based on an optical wedge consists of an autocollimation unit and an optical wedge. The autocollimation unit includes a light source, an autocollimation lens (Changchun Bena Optical Products Co., Ltd., Changchun, China), and two position-sensitive detectors (PSD1 and PSD2). The optical wedge is fixed to the measured target and is used to convert three-axis attitude variations into changes in the direction of the reflected beams. During the measurement process, the divergent beam emitted by the light source is collimated into a parallel beam by the autocollimation lens. When the beam is incident on the front surface of the optical wedge, part of it is refracted into the optical wedge and reflected by the rear surface, forming the primary path. Another part of the beam is directly reflected by the front surface, forming the secondary path. After being focused by the autocollimation lens, the two reflected beams form corresponding light spots at the focal plane. These spots carry the three-axis angular information of the optical wedge and are received by PSD1 and PSD2, respectively. The basic optical configuration and beam-propagation scheme were established in Ref. [10]. For the present study, Figure 1 is adapted from Ref. [10]. However, additional angular labels have been included in the present figure to facilitate the derivation of the analytical measurement-distance model.

When the optical wedge undergoes three-axis rotation together with the measured target, the centroid coordinates $\left(x_{1},\;y_{1}\right)$ and $\left(x_{2},\;y_{2}\right)$ of the primary- and secondary-path spots on the photosensitive surfaces of PSD1 and PSD2 shift accordingly. Accurate extraction and registration of the two spot coordinates are therefore required before subsequent measurement-distance determination and three-axis angle calculation. In practical measurement, the four-electrode outputs of each PSD are processed using a 34th-order finite impulse response (FIR) digital filter and converted into spot coordinates through normalized differential processing. The normalization by the total photocurrent reduces the influence of common-mode optical-intensity variations. Each PSD is calibrated for coordinate distortion, and the relative pose and position of the two PSDs are calibrated to transform the two coordinate outputs into a common reference frame.

To introduce the fixed-coefficient angle-solving model and facilitate formulation of the proposed distance–angle nested model, the initial angle-solving equations established in Ref. [10] are briefly restated below. Based on the principle of autocollimation imaging, and assuming that optical aberrations and multi-axis coupling effects are neglected, the initial values of pitch angle $\alpha_{p}$ and yaw angle $\beta_{p}$ can be expressed as:

(1) $$a_{p}=\frac{1}{2}\arctan\frac{y_{2}}{f}-\tau_{f},$$

(2) $$\beta_{p}=\frac{1}{2}\arctan\frac{x_{2}}{f},$$

In Equations (1) and (2), f is the focal length of the autocollimating lens, and $\tau_{f}$ is the angle between the front surface of the optical wedge and the XOY plane at the initial position.

Due to the geometric asymmetry of the optical wedge, variations in the roll angle γ primarily induce opposite displacements of the primary- and secondary-path spots along the *x*-axis. Therefore, the roll angle γ can be calculated using the *x*-coordinate difference in the two spots, and the initial value $\gamma_{p}$ is expressed as follows:

(3) $$\gamma_{p}=\frac{\left(x_{1}-x_{2}\right)}{2\times f\times n_{w}\times \sin\left(\tau_{f}-\tau_{b}\right)},$$

In Equation (3), $\tau_{b}$ is the angle between the rear surface of the optical wedge and the XOY plane at the initial position, and $n_{w}$ is the refractive index of the optical wedge material. In practical optical systems, optical aberrations and multi-axis coupling effects introduce systematic errors between the initial three-axis angles obtained from Equations (1)–(3) and the true attitude. To improve measurement accuracy, Ref. [10] calibrated the errors of the initial angle estimates using ternary polynomial regression and constructed a fixed-coefficient polynomial error-compensation model, as shown in Equation (4). For notational consistency, let $\varphi \in \left\{\alpha ,\;\beta ,\;\gamma \right\}$ denote the three-axis angle components. The corresponding angle-error compensation terms $\delta_{\varphi }$ can then be expressed as:

(4) $$\delta_{\varphi }=\sum_{i+j+k\leq n}h_{ijk}\alpha_{p}^{i}\beta_{p}^{j}\gamma_{p}^{k},$$

In Equation (4), $h_{ijk}$ denotes the polynomial coefficients; *i*, j, and k are non-negative integers; and n denotes the polynomial order. The final three-axis angles are obtained by subtracting the corresponding compensation terms from the initial values.

The fixed-coefficient polynomial error-compensation model described above can effectively correct systematic errors under fixed-distance conditions. It should be noted that the distance-induced measurement error originates from the change in the free-space propagation distance between the optical wedge and the autocollimation lens. The primary and secondary beams are generated through different optical paths of the optical wedge and therefore leave the wedge with different exit positions and propagation directions. When the optical wedge undergoes an axial displacement while its attitude remains unchanged, the propagation distance before the beams enter the autocollimation lens changes accordingly. Because the two beams propagate with nonzero off-axis angles, their incident heights at the autocollimation lens vary with the measurement distance. The autocollimation lens subsequently maps the incident beam heights and propagation angles to the PSD planes, causing the centroid coordinates of the primary and secondary spots to vary even when the actual attitude remains unchanged. The original fixed-coefficient angle-solving model does not include the measurement distance as an independent variable. Consequently, when the actual distance changes, the model interprets the distance-induced spot-coordinate shifts as attitude-induced coordinate variations, thereby producing distance-dependent nonlinear angle errors. To address this issue, the measurement distance *L* is introduced as an explicit variable into the fixed-coefficient angle-solving model so that the compensation coefficients can be updated according to the current measurement-distance condition, thereby forming the proposed distance–angle nested model.

### 2.2. Construction of the Analytical Measurement-Distance Model

To achieve high-precision angular measurements under varying-distance conditions, it is first necessary to separate the influence of measurement-distance variations from the coordinate information provided by the primary- and secondary-path spots. An analytical measurement-distance model is then established to provide real-time distance input for the subsequent distance–angle nested model. Geometrical-optics-based modeling has been employed in the design and optimization of compact optical sensing systems, such as miniaturized photoacoustic gas analyzers and multi-pass absorption-enhanced photoacoustic systems [12,13]. To this end, the ABCD ray-transfer matrix is used to model the beam propagation from the reference surface of the optical wedge (front surface) to the photosensitive surface of the PSDs. Let the exit height and propagation angle of the beam at the reference plane of the optical wedge be $y_{\mathrm{ref}}$ and $\theta_{\mathrm{ref}}$, and let the incident height and propagation angle at the photosensitive surface of the PSDs be $y_{\mathrm{PSD}}$ and $\theta_{\mathrm{PSD}}$. Then, the beam propagation relationship can be expressed as follows:

(5) $$\left[\begin{array}{c} y_{\mathrm{PSD}}\\ \theta_{\mathrm{PSD}} \end{array}\right]=\left[\begin{array}{cc} A(L) & B(L)\\ C(L) & D(L) \end{array}\right]\left[\begin{array}{c} y_{\mathrm{ref}}\\ \theta_{\mathrm{ref}} \end{array}\right],$$

**Figure 2:**
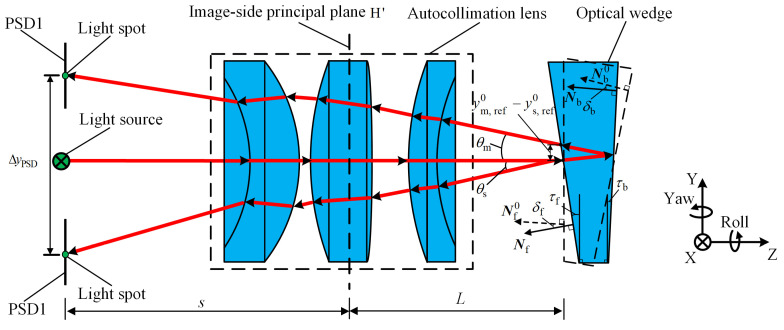
Geometric definition of the analytical measurement-distance model.

In Equation (5), A(L)=1−s/f, B(L)=L+s−Ls/f, C(L)=1/f, and D(L)=1−L/f, where s represents distance along the optical axis from the image-side principal plane $H^{\prime }$ of the autocollimation lens to the photosensitive surface of the PSDs, which coincides with the ideal focal plane of the optical system. It should be noted that the reflection and refraction processes at the front and rear surfaces of the optical wedge are determined using the vector forms of Snell’s law and the law of reflection. The ABCD ray-transfer formulation is employed only as a first-order equivalent model for the subsequent propagation of the returned primary and secondary beams through the autocollimation lens group to the PSD planes. To illustrate the geometric significance of each variable, a geometric model of the optical path was constructed, as shown in Figure 2. Here, $\Delta y_{\mathrm{PSD}}$ represents the difference in the *y*-coordinates of the primary and secondary spots on the photosensitive surface of the PSDs. $N_{f}^{0}$ and $N_{b}^{0}$ represent the normal vectors of the front and rear surfaces of the optical wedge in the initial state, respectively. The corresponding normal vectors after rotation are denoted by $N_{f}$ and $N_{b}$. $\delta_{f}$ and $\delta_{b}$ represent the angular changes in the normal vectors of the front and rear surfaces of the optical wedge relative to the initial state in the YOZ plane, respectively. $y_{m,\;\mathrm{ref}}^{0}$ and $y_{s,\;\mathrm{ref}}^{0}$ represent the exit heights of the primary and secondary beams at the reference plane of the optical wedge in the initial state, respectively. $\theta_{m}$ and $\theta_{s}$ denote the exit angles of the primary and secondary beams with respect to the optical axis in the YOZ plane at the reference plane of the optical wedge, respectively.

Due to the geometric characteristics of the optical wedge, when the measurement distance *L* varies, the primary- and secondary-path spots undergo approximately equal and opposite displacements, primarily along the *y*-axis. Based on this characteristic, the *y*-coordinate difference between the two spots is selected as the distance-sensitive feature. Compared with a single-spot coordinate, this characteristic quantity can effectively suppress the common-mode error introduced by optical aberrations and multi-axis coupling effects, making it more suitable for distance measurement under complex attitude conditions. By substituting the geometric parameters of the primary and secondary paths into the beam propagation model given in Equation (5), the analytical relationship between $\Delta y_{\mathrm{PSD}}$ and the measurement distance *L* is obtained, as shown in Equation (6):

(6) $$\Delta y_{\mathrm{PSD}}=(1-\frac{s}{f})\cdot (y_{m,\mathrm{ref}}^{0}-y_{s,\mathrm{ref}}^{0})+(L+s-\frac{Ls}{f})\cdot (\theta_{m}-\theta_{s}),$$

As indicated in Equation (6), $\Delta y_{\mathrm{PSD}}$ consists of two components. The first component is the difference between the initial exit heights $y_{m,\;\mathrm{ref}}^{0}$ and $y_{s,\;\mathrm{ref}}^{0}$ of the primary and secondary beams, which is determined jointly by the wedge angle, central thickness, and refractive index of the optical wedge, and can be regarded as a constant after the structural parameters of the optical system are determined. In this system, $y_{m,\;\mathrm{ref}}^{0}$ and $y_{s,\;\mathrm{ref}}^{0}$ are 0.721 mm and 0 mm, respectively. The second component is the difference between the exit angles $\theta_{m}$ and $\theta_{s}$ of the primary and secondary beams, which is affected by both the attitude of the optical wedge and the measurement distance. Therefore, determining the measurement distance *L* can be transformed into a modeling problem of the exit angles $\theta_{m}$ and $\theta_{s}$.

For the primary beam, the changes in the propagation angle at the front and rear surfaces can be derived sequentially according to Snell’s law and the law of reflection. The expression for the exit angle $\theta_{m}$ is then obtained as follows:

(7) $$\theta_{m}=\left(\tau_{f}+\delta_{f}\right)+\arcsin\left[n_{w}\cdot \sin\left(2\left(\tau_{b}-\tau_{f}\right)+2\left(\delta_{b}-\delta_{f}\right)+\arcsin\left(\frac{1}{n_{w}}\sin\left(\tau_{f}+\delta_{f}\right)\right)\right)\right].$$

Similarly, for the secondary beam directly reflected by the front surface of the optical wedge, the expression for its exit angle $\theta_{s}$ is given in Equation (8):

(8) $$\theta_{s}=2\left(\tau_{f}+\delta_{f}\right),$$

In Equations (7) and (8), $\delta_{f}$ and $\delta_{b}$ are directly related to the three-axis attitude of the optical wedge and are obtained by applying an Euler rotation transformation. Let the normal vectors of the front and rear surfaces of the optical wedge in the initial state be $N_{f}^{0}={\left[0,\;-\sin\tau_{f},\;-\cos\tau_{f}\right]}^{T}$ and $N_{b}^{0}={\left[0,\;\sin\tau_{b},\;-\cos\tau_{b}\right]}^{T}$. After the three-axis rotation, the corresponding normal vectors are transformed into $N_{f}$ and $N_{b}$. The transformation from the initial normal vectors to the rotated normal vectors can be expressed as:

(9) $$\left\{\begin{array}{l} N_{f}=R_{t}\cdot N_{f}^{0}\\ N_{b}=R_{t}\cdot N_{b}^{0} \end{array}\right.,$$

In Equation (9), $R_{t}$ denotes the rotation matrix composed of the pitch angle α, yaw angle β, and roll angle γ, and is expressed as follows:

(10) $$R_{t}=\left[\begin{array}{ccc} 1 & 0 & 0\\ 0 & \cos\alpha & -\sin\alpha \\ 0 & \sin\alpha & \cos\alpha \end{array}\right]\cdot \left[\begin{array}{ccc} \cos\beta & 0 & \sin\beta \\ 0 & 1 & 0\\ -\sin\beta & 0 & \cos\beta \end{array}\right]\cdot \left[\begin{array}{ccc} \cos\gamma & -\sin\gamma & 0\\ \sin\gamma & \cos\gamma & 0\\ 0 & 0 & 1 \end{array}\right].$$

Combining Equations (9) and (10), the *y*- and *z*-components of $N_{f}$ and $N_{b}$ in the YOZ plane can be obtained, as shown in Equations (11) and (12):

(11) $$\left\{\begin{array}{l} N_{f,\;y}=-\cos\alpha \cos\gamma \sin\tau_{f}+\sin\alpha \left(\sin\beta \sin\gamma \sin\tau_{f}+\cos\beta \cos\tau_{f}\right)\\ N_{f,\;z}=-\sin\alpha \cos\gamma \sin\tau_{f}-\cos\alpha \left(\sin\beta \sin\gamma \sin\tau_{f}+\cos\beta \cos\tau_{f}\right) \end{array}\right.,$$

(12) $$\left\{\begin{array}{l} N_{b,\;y}=\cos\alpha \cos\gamma \sin\tau_{b}+\sin\alpha \left(\cos\beta \cos\tau_{b}-\sin\beta \sin\gamma \sin\tau_{b}\right)\\ N_{b,\;z}=\sin\alpha \cos\gamma \sin\tau_{b}-\cos\alpha \left(\cos\beta \cos\tau_{b}-\sin\beta \sin\gamma \sin\tau_{b}\right) \end{array}\right.,$$

From Equations (11) and (12), the expressions for $\delta_{f}$ and $\delta_{b}$ are obtained, as given in Equation (13):

(13) $$\left\{\begin{array}{l} \delta_{f}=\arctan(\frac{N_{f,\;y}^{\prime }}{N_{f,\;z}^{\prime }})-\tau_{f}\\ \delta_{b}=\arctan(\frac{N_{b,y}^{\prime }}{N_{b,\;z}^{\prime }})-\tau_{b} \end{array}\right..$$

After combining Equations (6)–(13), an intermediate variable $\Theta_{m}$ is introduced to improve the readability of the expression. This variable represents the attitude-dependent term in the exit-angle difference between the primary and secondary beams, as given in Equation (14):

(14) $$\Theta_{m}=\left(\tau_{f}+\delta_{f}\right)-\arcsin\left\{n_{w}\cdot \sin\left[2\left(\tau_{b}-\tau_{f}\right)+2\left(\delta_{b}-\delta_{f}\right)+\arcsin\left(\frac{1}{n}\sin\left(\tau_{f}+\delta_{f}\right)\right)\right]\right\}.$$

The analytical measurement-distance model can then be expressed as Equation (15):

(15) $$L=\frac{\Delta y_{\mathrm{PSD}}-(1-s/f)(y_{m,\;\mathrm{ref}}^{0}-y_{s,\;\mathrm{ref}}^{0})}{(1-s/f)\Theta_{m}}-\frac{s}{1-s/f}.$$

This model enables *L* to be directly determined from the coordinate information of the two spots. As a result, *L* is no longer treated as an implicit error term in the fixed-coefficient angle-solving model. Instead, it serves as an explicit variable in the subsequent distance–angle nested compensation procedure. Because the two returned beams are intentionally directed toward off-axis PSD positions, the complete optical system is not treated as globally paraxial. In addition, the residual error of the first-order analytical measurement-distance model cannot be uniquely attributed to the paraxial approximation alone because it is coupled with residual off-axis imaging effects, lens aberrations, multi-axis coupling effects, and other simplifications in the equivalent lens representation. Therefore, the validity of the proposed analytical measurement-distance model is evaluated in Section 3.1 using a non-paraxial ray-tracing model as the numerical reference.

### 2.3. Establishment of the Distance–Angle Nested Model

To overcome the inability of the fixed-coefficient angle-solving model to effectively compensate for systematic errors under varying-distance conditions, this section extends the coefficient $h_{ijk}$ in the fixed-coefficient polynomial error-compensation model from a constant to a function of *L*. A distance–angle nested model is thereby constructed, allowing the model coefficients to be updated in real time as the measurement distance varies.

First, ray tracing is performed based on the optical simulation model to obtain the three-axis attitude angles $\left(\alpha ,\;\beta ,\;\gamma \right)$ of the optical wedge under different measurement-distance conditions, along with the corresponding coordinates of the primary and secondary spots, $\left(x_{1},y_{1}\right)$ and $\left(x_{2},y_{2}\right)$. On this basis, ternary polynomial error-compensation models are constructed for each measurement-distance condition, and the fixed compensation coefficients are expressed as *m*-th-order polynomial functions of *L*, namely:

(16) $$h_{ijk}=\sum_{q=0}^{m}c_{ijk}^{\left(q\right)}L^{q},$$

In Equation (16), $c_{ijk}^{\left(q\right)}$ denotes the *q*-th-order expansion coefficient of $h_{ijk}$ with respect to *L*, q is a non-negative integer, and m denotes the order of the distance polynomial. Substituting Equation (16) into the fixed-coefficient angle-error compensation term $\delta_{\varphi }$ yields the corresponding distance-dependent angle-error compensation term $\delta_{\varphi }(L)$:

(17) $$\delta_{\varphi }(L)=\sum_{i+j+k\leq n}\sum_{q=0}^{m}c_{ijk}^{\left(q\right)}L^{q}\alpha_{p}^{i}\beta_{p}^{j}\gamma_{p}^{k}.$$

Therefore, the final form of the distance–angle nested model can be expressed as:

(18) $$\varphi_{\mathrm{corr}}=\varphi_{p}-\delta_{\varphi }(L).$$

In the present implementation, the ternary polynomial orders *n* used for the pitch-, yaw-, and roll-angle error-compensation models in Equation (17) were 4, 5, and 5, respectively. The distance-polynomial order *m* was set to 3 for all three axes. Thus, the coefficient associated with each term in the ternary polynomial was represented as a third-order polynomial function of the measurement distance *L*.

The values of *n* and *m* were determined by comparing candidate models with different ternary-polynomial orders and distance-polynomial orders. Increasing the orders from lower-order candidates to the selected values resulted in a clear improvement in prediction accuracy. Further increases in either *n* or *m* produced only marginal additional improvements, while substantially increasing the number of polynomial terms, model complexity, storage requirements, and real-time computational burden. Therefore, the selected values of *n* and *m* were adopted by considering prediction accuracy, generalization ability, and computational efficiency. During the polynomial-fitting process, the 729 simulated attitude samples at each measurement-distance condition were randomly divided into an 80% calibration subset and a 20% held-out test subset. Only the calibration subset was used to determine the coefficients of the ternary polynomials at each measurement-distance condition and to fit the corresponding distance-dependent coefficient functions. The remaining 20% of samples were excluded from all coefficient-fitting procedures and retained for evaluating the prediction performance on unseen attitude combinations.

Based on the foregoing model, the computational procedure comprises an initial-angle calculation, measurement-distance determination, coefficient updating, and final angle correction. The initial angles are first calculated using the fixed-coefficient angle-solving model. The measurement distance *L* is then determined from the centroid coordinates of the primary- and secondary-path spots and substituted into the distance–angle nested model to update the compensation coefficients. The corrected three-axis angles are finally obtained. Through real-time feedback of the measurement distance, the compensation coefficients are adaptively updated, thereby improving measurement accuracy under varying-distance conditions. The compensation coefficients of the proposed distance–angle nested model are specific to the optical configuration and alignment condition used in this study. When the optical wedge parameters, lens configuration, PSD arrangement, or alignment condition change significantly, the geometrical parameters used in the analytical measurement-distance model and the compensation coefficients of the distance–angle nested model should be updated.

## 3. Simulation Verification of the Distance-Adaptive Method for Three-Axis Angle Measurement

**Figure 3:**
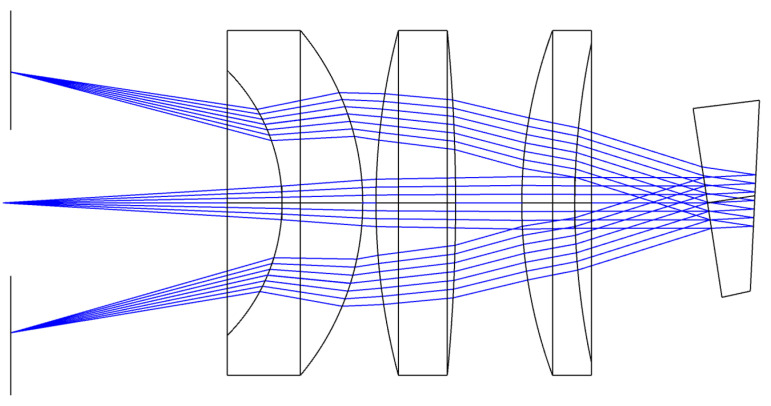
Two-dimensional cross-sectional schematic of the optical simulation model (blue lines: traced rays; black lines: optical-component outlines).

To verify the effectiveness of the proposed distance-adaptive method for the three-axis angle measurement, an optical simulation model simultaneously incorporating variations in the three-axis attitude of the optical wedge and the measurement distance was established, as shown in Figure 3. Based on this model, the accuracy of the analytical measurement-distance model and its stability against the coupled variations in the three-axis attitude are first analyzed. The error-compensation performance of the proposed method is then evaluated under conditions involving variations in both the measurement distance and the optical wedge attitude.

### 3.1. Simulation Analysis of the Accuracy of the Analytical Measurement-Distance Model

**Figure 4:**
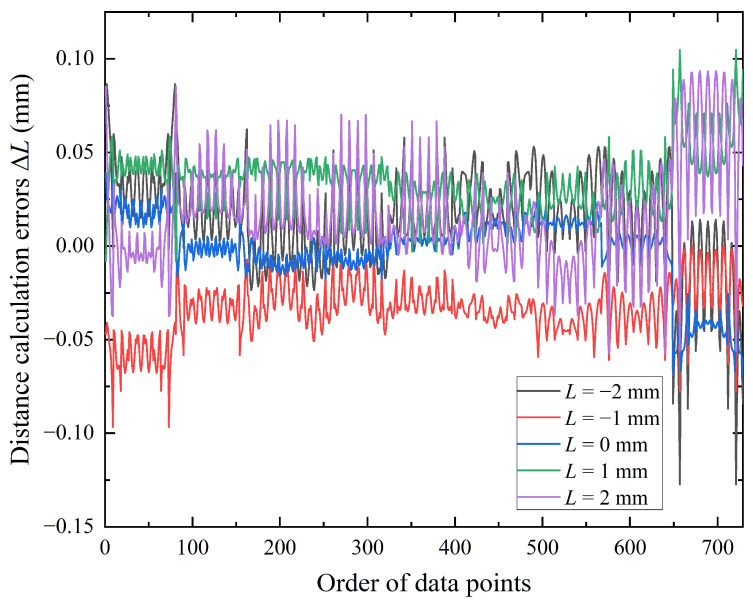
Distribution of measurement-distance errors under full-factorial attitude conditions.

To verify the accuracy of the analytical measurement-distance model and its stability under the condition of three-axis attitude coupling, a simulation analysis was conducted over the full-factorial attitude space. In the simulation, five measurement-distance conditions, namely *L* = −2 mm, −1 mm, 0 mm, +1 mm, and +2 mm, were selected to cover the axial displacement range of the inner ring frame of the gimbal platform. At each measurement-distance condition, a full-factorial scan of the three-axis attitude angles of the optical wedge was performed within the range of [−2°, +2°] with a step size of 0.5°, thereby generating 729 attitude samples. The centroid coordinates of the light spots formed by the primary and secondary beams were obtained by ray tracing. These coordinates were then substituted into the analytical measurement-distance model. The resulting distribution of the measurement-distance errors under the full-factorial attitude conditions is shown in Figure 4.

As shown in Figure 4, the error curves under different measurement-distance conditions exhibit generally consistent trends, differing primarily in magnitude. This indicates that variations in measurement distance mainly affect the error magnitude, while having a relatively small influence on the distribution pattern of the error with respect to three-axis attitude variations. Further statistical analysis shows that, for *L* = −2 mm, −1 mm, 0 mm, +1 mm, and +2 mm, the RMS errors are 0.0308 mm, 0.0368 mm, 0.0191 mm, 0.0375 mm, and 0.0312 mm, respectively. The maximum absolute errors are all less than 0.127 mm. The results indicate that, within the specified ranges of measurement distance and attitude angles, the established model can provide stable and reliable distance input for the subsequent distance–angle nested model. It should be noted that the errors shown in Figure 4 represent the overall discrepancy between the proposed first-order analytical measurement-distance model and the complete non-paraxial ray-tracing reference. These errors include the combined effects of the first-order approximation, residual off-axis imaging effects, lens aberrations, multi-axis coupling effects, and other simplifications in the analytical formulation. Therefore, within the sampled operating range, the results in Figure 4 provide a conservative system-level estimate of the residual modeling error of the proposed analytical measurement-distance model rather than an error caused exclusively by the paraxial approximation.

### 3.2. Simulation Verification of Angular Error Compensation Performance

To verify the error-compensation performance of the proposed distance-adaptive method, the angular errors before and after compensation are compared under conditions involving variations in both the measurement distance and the optical wedge attitude.

**Figure 5:**
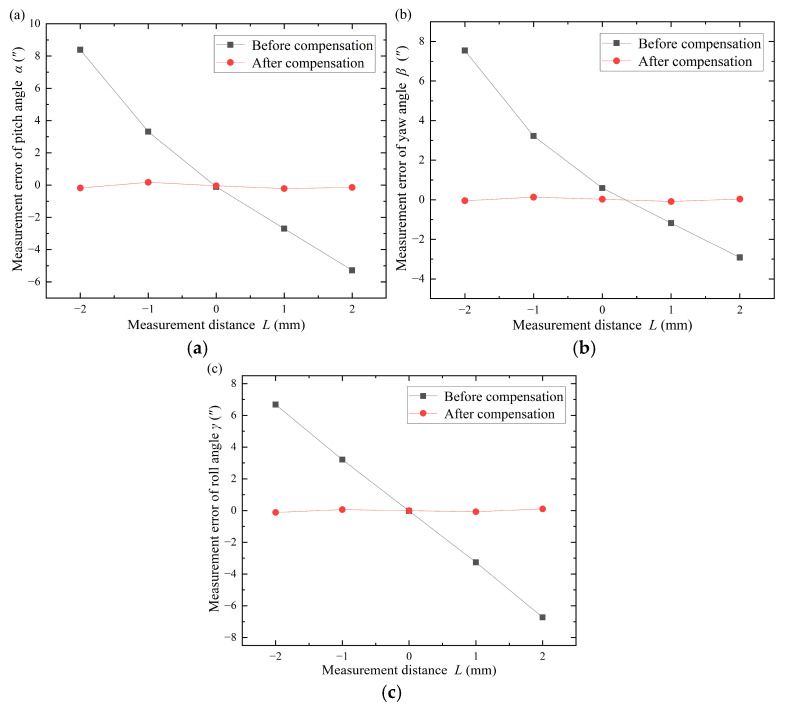
Relationship between angular error and measurement distance *L* before and after compensation: (**a**) pitch angle α; (**b**) yaw angle β; (**c**) roll angle γ.

To analyze the effect of measurement-distance variations on angular errors, *L* was varied from −2 mm to +2 mm with a step size of 1 mm while the attitude of the optical wedge was kept fixed, and the three-axis angular errors before and after compensation were evaluated. Typical single-axis-dominant attitude conditions were selected for the optical wedge. Specifically, the conditions for analyzing the errors of the pitch angle α, yaw angle β, and roll angle γ were set to $\left(\alpha ,\;\beta ,\;\gamma \right)=\left(-2,\;0,\;0\right)$, $\left(0,\;-2,\;0\right)$, and $\left(0,\;0,\;-2\right)$, respectively. The relationship between the angular error and the measurement distance before and after compensation is shown in Figure 5.

As shown in Figure 5, before compensation, the three-axis angular errors exhibit an approximately monotonic trend with measurement distance, with a maximum error of about 8.4″, indicating pronounced distance dependence. After compensation, the errors are significantly reduced and remain within ±0.2″. These results demonstrate that the proposed method effectively reduces the sensitivity of the angular errors to measurement distance.

**Figure 6:**
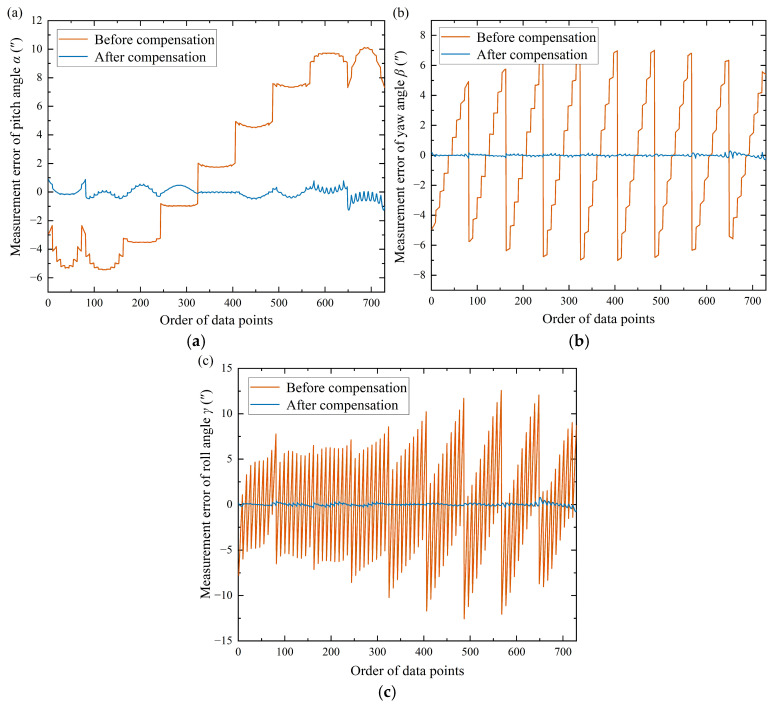
Comparison of the distribution characteristics of three-axis angular errors before and after compensation at the relative measurement-distance condition of *L* = +2 mm: (**a**) pitch angle α; (**b**) yaw angle β; (**c**) roll angle γ.

To further verify the compensation capability of the proposed method under varying optical wedge attitude conditions, the maximum relative measurement-distance condition, L=+2 mm, was selected as a representative condition. At this distance, a full-factorial scan was performed over the three-axis attitude space within the range of [−2°, +2°] with a step size of 0.5°, generating 729 attitude samples. The three-axis angular errors before and after compensation were calculated for all 729 samples, and the results are shown in Figure 6. The full-attitude simulation results shown in Figure 6 include both the calibration samples and the held-out test samples, thereby characterizing the overall performance of the proposed method throughout the complete three-axis attitude space.

As shown in Figure 6, at the maximum relative measurement-distance condition, *L* = +2 mm, the three-axis angular errors before compensation fall within approximately ±13″ across the full-factorial attitude space and exhibit pronounced fluctuations with changes in the optical wedge attitude. After compensation, the error range is reduced to approximately ±1.5″, and the error distribution becomes significantly smoother. This indicates that the proposed method remains effective in suppressing distance-dependent systematic errors under the coupled effects of measurement distance and multi-axis attitude, thereby enhancing the model’s adaptability to complex operating conditions. It should be noted that the residual fluctuation patterns after compensation are not caused by numerical instability, since similar characteristics are also present in the uncompensated results. The distance–angle nested compensation procedure is implemented as a one-pass algebraic calculation without iterative feedback. Although the distance–angle nested model effectively suppresses the systematic errors induced by measurement-distance variations, the remaining fluctuations mainly arise from finite-order approximation residuals and higher-order cross-axis coupling effects. These effects are more pronounced for roll because it is determined primarily from the differential *x*-coordinate feature of the two spots, which has relatively low sensitivity to roll-angle variation.

## 4. Experimental Results and Discussion

**Figure 7:**
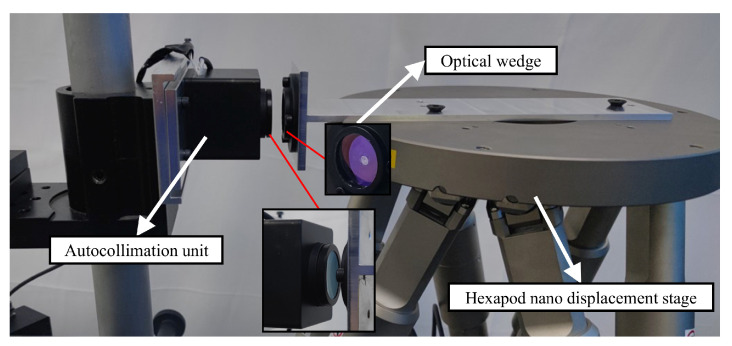
Experimental system.

To verify the feasibility and error-compensation performance of the proposed method in a practical measurement system, the experimental platform established in Ref. [10] was employed in the present study. Figure 7 shows the same platform, with an enlarged view of the autocollimation unit for clearer visualization. The system consists of an autocollimation measurement unit, an optical wedge, an optical wedge mount, and a hexapod nano displacement stage. The optical wedge mount is rigidly connected to the hexapod nano displacement stage via a connector, allowing the optical wedge to rotate and translate along three axes. In the autocollimation measurement unit, a three-element autocollimation lens was employed. Its nominal effective focal length in the optical design model was 31.956 mm. The experimental lens was fabricated according to the same optical prescription. The two PSDs are PSD100-IC devices manufactured by Shanghai Ouguang Electronics (Shanghai, China). Each PSD has a photosensitive area of 9 mm×9 mm, a spectral response range of 400–1100 nm, a dark current of 50 nA, and a positional resolution of 1.5 μm. The light source was a high-power vertical-cavity surface-emitting laser (VCSEL), ING-2835VCSELIR940CB01, manufactured by Shenzhen Bright Photon Technology Co., Ltd. (Shenzhen, China), with a wavelength of 940 nm and an output power of 3 mW. Combined with a pinhole aperture integrated into the prototype, the laser produced a beam with a divergence angle of approximately 12.6°. An H850-PI hexapod nano displacement stage (Physik Instrumente (PI) SE & Co. KG, Karlsruhe, Germany) was used. Along the *X*, *Y*, and *Z* axes, its rotational ranges were ±15°, ±15°, and ±30°, respectively; the rotational resolutions were 3 μrad, 3 μrad, and 5 μrad, respectively; and the rotational accuracies were ±3 μrad, ±3 μrad, and ±9 μrad, respectively. The translational ranges along the *X*, *Y*, and *Z* axes were ±50 mm, ±50 mm, and ±30 mm, respectively; the translational resolutions were 0.3 μm, 0.3 μm, and 0.2 μm, respectively; and the translational accuracies were ±0.6 μm, ±0.6 μm, and ±0.2 μm, respectively. The stage drove the optical wedge to generate coupled three-axis attitude and axial-displacement motions, while also serving as the reference standard for angle and displacement inputs.

To reduce the influence of alignment errors on the experimental results, the PSD coordinate system was aligned with the measurement system coordinate system before the experiment so that the corresponding coordinate axes were parallel. The specific calibration method is detailed in Ref. [10]. The experimental measurements described in this section were not used in constructing the proposed distance–angle nested model. Instead, the model was constructed solely from the ray-tracing simulation data, whereas the experimental measurements were used only to evaluate the proposed method under practical measurement conditions.

**Figure 8:**
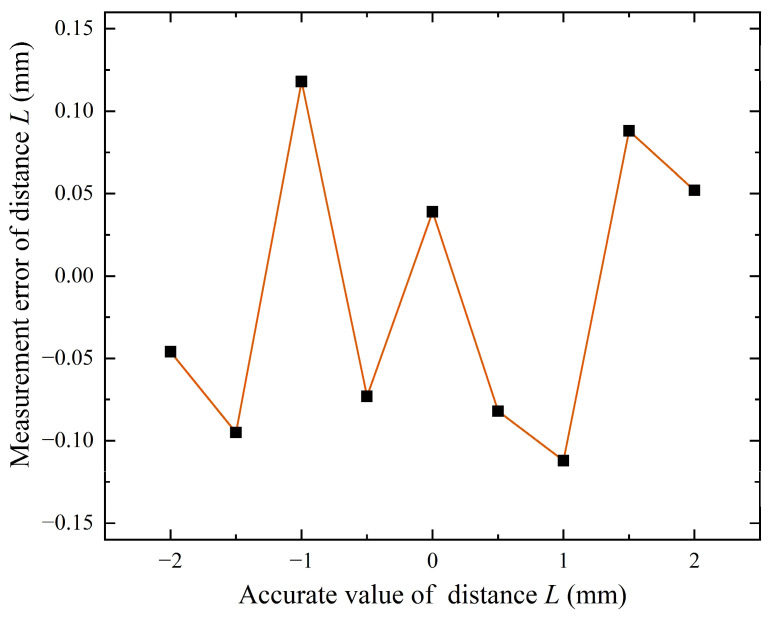
Measurement-distance errors at different measurement distances.

Accurate determination of measurement distance *L* is a prerequisite for the effective implementation of the distance-adaptive method for the three-axis angle measurement. Therefore, the established analytical measurement-distance model was first experimentally verified. In the experiment, the attitude angles of the optical wedge were fixed at (α,β,γ)=(0°, 0°, 0°), and the relative axial displacement was varied from L=−2 mm to L=+2 mm with a step size of 0.5 mm, yielding a total of nine measurement points. With 10 mm as the nominal measurement distance, these conditions correspond to actual physical working distances from 8 mm to 12 mm. This range was selected according to the engineering operating requirement specified for the target airborne optoelectronic gimbal prototype. The measurement distance *L* was determined from the centroid coordinates of the two spots and then compared with the actual input values of the nano displacement stage. The difference between them was defined as the measurement-distance error. Each measurement point was independently measured six times, and the mean of the six error values was used as the final result to reduce the influence of random noise. The errors at different measurement distances are shown in Figure 8.

As shown in Figure 8, within the measurement range of ±2 mm, the RMS value of the distance error is 0.09 mm. The experimental results indicate that the established analytical measurement-distance model can provide stable and accurate distance inputs for the subsequent distance–angle nested model.

After the accuracy of measurement-distance determination was verified, *L* was further introduced into the distance–angle nested model to evaluate the compensation performance of the proposed method for three-axis angular errors. The boundary relative measurement-distance conditions (*L* = ±2 mm) and the zero position (*L* = 0 mm) were selected as typical conditions, and the three-axis angular errors were evaluated independently using a single-axis scanning method. Taking the pitch angle α as an example, the yaw angle β and roll angle γ were kept at 0°, while α was varied from −2° to +2° in 0.5° increments. The output values of the autocollimation measurement unit were recorded and compared with the actual inputs of the nano displacement stage, and their difference was taken as the pitch-angle error. This process was repeated six times, and the mean of the six differences was used as the final error value. The measurement errors of the yaw angle β and roll angle γ were evaluated in the same manner.

**Figure 9:**
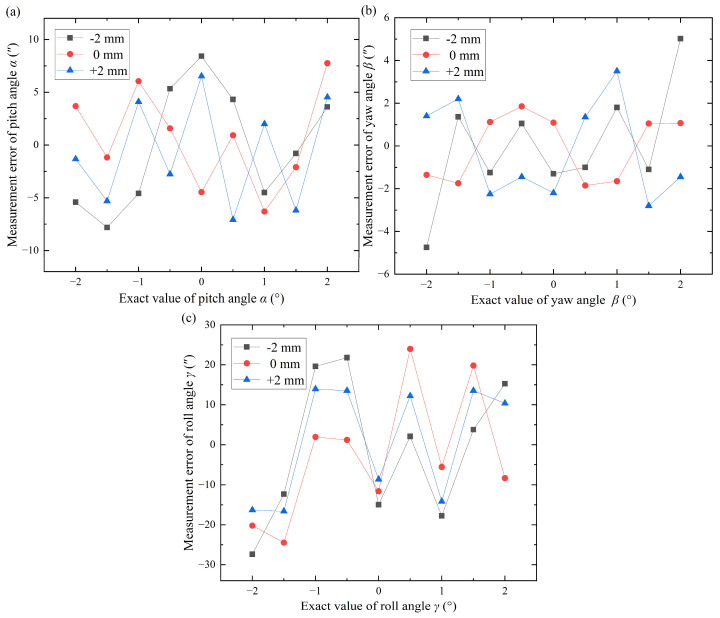
Three-axis angle measurement errors at different measurement distances: (**a**) pitch angle α; (**b**) yaw angle β; (**c**) roll angle γ.

As shown in Figure 9, after applying the distance–angle nested compensation procedure, the three-axis angular errors under different measurement-distance conditions remained within a relatively small range, indicating that the system achieved good measurement accuracy and consistency. The three physical measurement-distance conditions considered in this study are identical to those reported in Ref. [10], namely, 8 mm, 10 mm, and 12 mm. In the present study, the 10 mm condition is selected as the initial reference distance, and the relative measurement distance is defined as L=D−10 mm, where *D* denotes the physical measurement distance. Therefore, the physical distances of 8 mm, 10 mm, and 12 mm correspond to L=−2 mm, L=0 mm, and L=+2 mm, respectively. The fixed-coefficient RMS angular errors used as baseline results in the following comparison were reported in Ref. [10]. They are cited here solely to evaluate the improvement achieved by the proposed method. The corresponding RMS angular errors in the present study were obtained using the distance–angle nested compensation procedure based on the distance–angle nested model under the same optical configuration, experimental platform, and matched measurement-distance conditions. Compared with the fixed-coefficient baseline results reported in Ref. [10], the RMS errors of the pitch angle α at the relative measurement-distance conditions of L=−2 mm, L=0 mm, and L=+2 mm decreased from 12.1″, 7.8″, and 10.7″ to 5.4″, 4.5″, and 4.8″, respectively. For the yaw angle β, the RMS errors decreased from 6.5″, 1.5″, and 3.3″ to 2.6″, 1.5″, and 2.2″, respectively. For the roll angle γ, the RMS errors decreased from 28.4″, 22.2″, and 15.6″ to 16.8″, 15.7″, and 13.5″, respectively.

To further evaluate the performance of the proposed method under coupled three-axis attitudes, additional experiments were conducted under three relative measurement-distance conditions: L=−2 mm, L=0 mm, and L=+2 mm. Sixteen representative attitude combinations with simultaneously nonzero pitch, yaw, and roll angles were tested at each relative measurement-distance condition. At L=−2 mm, the RMS errors of pitch, yaw, and roll decreased from 13.0″, 8.4″, and 31.1″ to 5.6″, 3.8″, and 18.6″, respectively, after applying the distance–angle nested compensation procedure. At L=0 mm, the corresponding RMS errors decreased from 8.0″, 2.1″, and 24.9″ to 5.2″, 2.0″, and 17.6″, respectively. At L=+2 mm, the RMS errors decreased from 11.6″, 4.5″, and 20.3″ to 5.1″, 2.6″, and 15.0″, respectively. These results indicate that the proposed distance-adaptive method can reduce the overall three-axis RMS errors under coupled attitude conditions at all tested relative measurement distances. Therefore, the measurement-distance determination and distance–angle nested compensation procedure remain effective when pitch, yaw, and roll occur simultaneously. The detailed pointwise errors for all coupled attitude combinations are provided in Supplementary Tables S1–S6. These results satisfy the target requirements of 0.01° for pitch and yaw and 0.1° for roll. Although the residual roll error is higher than the pitch and yaw errors, it remains well below the required roll-angle accuracy. This difference is mainly attributed to the lower sensitivity of the dual-PSD differential *x*-direction displacement to roll, which makes the recovered roll angle more susceptible to residual centroid, alignment, and cross-axis coupling errors.

The experimental results indicate that the proposed distance-adaptive method can effectively suppress the measurement errors caused by axial displacement, thereby reducing the sensitivity of the angular results to measurement distance. It should be noted that the roll angle γ exhibited a relatively large error before compensation, primarily because the *x*-axis coordinate difference between the two spots has low sensitivity to roll-angle variations. As a result, the roll-angle measurement is more susceptible to PSD noise, centroid extraction errors, and system alignment errors. After applying the distance–angle nested compensation procedure, the roll-angle error was significantly reduced, demonstrating that the proposed method remains effective even in the low-sensitivity direction. In addition, under laboratory conditions, the proposed three-axis angle-measurement sensor was integrated with the target airborne optoelectronic gimbal platform. The measured overall update period of the complete measurement-and-compensation cycle was 963 μs, corresponding to an update rate of approximately 1038 Hz, which satisfies the update-rate requirement of the target platform.

## 5. Conclusions

The three-axis angle measurement system based on an optical wedge exhibits pronounced sensitivity to measurement distance under short-focal-length conditions. When the measurement distance varies, the accuracy of the fixed-coefficient angle-solving model decreases significantly. To address this problem, this study proposes a distance-adaptive method for three-axis angle measurements. Based on ABCD ray-transfer-matrix theory, the method establishes an analytical measurement-distance model and introduces *L* as an explicit variable into the fixed-coefficient angle-solving model, allowing the fixed compensation coefficients to be updated in real time with distance and thereby suppressing distance-induced measurement errors.

The experimental results demonstrate that, over the measurement-distance range of −2 mm to +2 mm, the established analytical measurement-distance model achieves an RMS error of 0.09 mm, providing reliable distance input for the distance–angle nested model. After applying the distance–angle nested compensation procedure, the three-axis angular errors at different measurement distances were significantly reduced. Taking the measurement-distance condition corresponding to the maximum error, *L* = −2 mm, as an example, the RMS errors of the pitch angle α, yaw angle β, and roll angle γ decreased from 13.0″, 8.4″, and 31.1″ to 5.6″, 3.8″, and 18.6″, respectively.

The results demonstrate that the proposed distance-adaptive method for the three-axis angle measurement can improve the measurement accuracy and consistency of the system under varying-distance conditions without requiring additional sensors or increasing the system size. Therefore, this method provides a feasible technical solution for high-accuracy attitude feedback in fifth-generation airborne optoelectronic gimbal platforms, and also offers a new implementation approach for non-contact three-axis angle measurements under other space-constrained conditions. The present laboratory experiments primarily validate the proposed method under static and quasi-static conditions. The sensor was subsequently integrated with the target airborne optoelectronic gimbal platform by a collaborating research institute and verified under representative laboratory operating conditions, including dynamic motion, vibration, thermal drift, and other practical operational factors. The integrated system satisfied the relevant project requirements. Nevertheless, actual flight testing after installation on an aircraft has not yet been completed and will be conducted in future work. Future work will focus on dynamic measurement and thermal-drift compensation to further improve the applicability of the proposed method in complex engineering environments. In particular, the effects of spot size, shape, and temporal stability on PSD coordinate uncertainty and its propagation to measurement-distance and angle errors will be quantitatively investigated.

## Data Availability

The raw data supporting the conclusions of this article will be made available by the authors on request.

## Supplementary Materials

The following supporting information can be downloaded at: https://www.mdpi.com/article/10.3390/s26144430/s1, Table S1: Three-axis angular errors under coupled attitude conditions before and after distance compensation at *L* = 0 mm; Table S2: Comparison of RMS three-axis angular errors under coupled attitude conditions before and after distance compensation at *L* = 0 mm; Table S3: Three-axis angular errors under coupled attitude conditions before and after distance compensation at *L* = −2 mm; Table S4: Comparison of RMS three-axis angular errors under coupled attitude conditions before and after distance compensation at *L* = −2 mm; Table S5: Three-axis angular errors under coupled attitude conditions before and after distance compensation at *L* = +2 mm; Table S6: Comparison of RMS three-axis angular errors under coupled attitude conditions before and after distance compensation at *L* = +2 mm.

## References

[1] Lan L., Jiang W., Hua F. (2023). Research on the line of sight stabilization control technology of optronic mast under high oceanic condition and big swaying movement of platform. Sensors.

[2] Wang L., Li X., Zhou Z., Liu Y., Yang Z., Zhang S., Li C. (2024). Disturbance observation and suppression in an airborne electro-optical stabilized platform based on a generalized high-order extended state observer. Sensors.

[3] Qi Y., Wang H.L., Xu Q.Q., Du Y.L., Shao X.Z., Yang H. (2021). A new flexible gimbal for electro-optical sighting system (EOSS). Optik.

[4] Gao W., Saito Y., Muto H., Arai Y., Shimizu Y. (2011). A three-axis autocollimator for detection of angular error motions of a precision stage. CIRP Ann..

[5] Yin Y., Cai S., Qiao Y. (2016). Design, fabrication, and verification of a three-dimensional autocollimator. Appl. Opt..

[6] Li R., Zhen Y., Di K., Wang W., Nikitin M., Tong M.H., Zhang Y., Zou X., Konyakhin I. (2021). Three-degree-of-freedom autocollimator with large angle-measurement range. Meas. Sci. Technol..

[7] Guo Y., Cheng H., Liu G. (2023). Three-degree-of-freedom autocollimation angle measurement method based on crosshair displacement and rotation. Rev. Sci. Instrum..

[8] Li X., Shimizu Y., Ito T., Cai Y., Ito S., Gao W. (2014). Measurement of six-degree-of-freedom planar motions by using a multiprobe surface encoder. Opt. Eng..

[9] Hsieh H.-L., Pan S.-W. (2015). Development of a grating-based interferometer for six-degree-of-freedom displacement and angle measurements. Opt. Express.

[10] Wang J., Bai Y., Lu H., Wang Y., Zhang L. (2025). Lightweight and compact three-axis angle measurement method based on an optical wedge. Opt. Express.

[11] Siegman A.E. (1986). Lasers.

[12] Zhang G., Guo M., Zhao X., Cui D., Xu L., Wang N., Ni R., Qi H., Zhao J., Chen K. (2023). Miniaturized Nonresonant Photoacoustic Gas Analyzer for CO_2_ Detection. Microw. Opt. Technol. Lett..

[13] Qi H., Zhao X., Li C., Zhang Z., Zhang G., Chen K. (2026). Multi-Pass Absorption Enhanced Photoacoustic System for Humidity-Independent Atmospheric CO Detection. Sens. Actuators B Chem..

