# Supplementary material for: A Distance-Adaptive Method for Three-Axis Angle Measurement Based on an Optical Wedge
Source: Sensors (Basel). 2026 Jul 12;26(14):4430. doi: 10.3390/s26144430 (PMC13416873; doi:10.3390/s26144430)
Supplement: Supplementary file 1 [file sensors-26-04430-s001.zip › sensors-4373559-supplementary.pdf]

Table S1. Three-axis angular errors under coupled attitude conditions before and after distance compensation at $L = 0$ mm .

Case	α / (°)	β / (°)	γ / (°)	Before compensation			After compensation		
				$\Delta\alpha$ / (″)	$\Delta\beta$ / (″)	$\Delta\gamma$ / (″)	$\Delta\alpha$ / (″)	$\Delta\beta$ / (″)	$\Delta\gamma$ / (″)
1	−2	−2	−2	8.9	2.8	33.6	3.2	−2.3	21.5
2	−2	−1	−1	7.7	0.9	14.2	2.9	1.5	16.5
3	−2	+1	+1	7.9	−2.1	−17.4	3.0	−3.0	−12.8
4	−2	+2	+2	9.3	−3.5	−37.9	3.4	0.9	−24.5
5	−1	−2	−1	10.1	2.3	15.6	5.0	−1.9	11.4
6	−1	−1	+1	9.3	0.6	−13.9	4.6	1.2	−16.0
7	−1	+1	+2	10.4	−2.0	−30.6	5.1	−3.0	−17.1
8	−1	+2	−2	12.0	−2.3	29.0	5.8	1.7	17.8
9	+1	−2	+1	6.2	2.4	−13.0	−5.7	−1.7	−15.5
10	+1	−1	+2	6.3	1.1	−27.7	−5.6	1.5	−18.5
11	+1	+1	−2	7.4	−0.8	28.5	−5.0	−1.9	19.4
12	+1	+2	−1	7.5	−1.7	14.7	−5.0	1.7	17.0
13	+2	−2	+2	5.0	3.1	−32.1	6.6	−2.2	−21.6
14	+2	−1	−2	5.4	2.2	33.4	6.6	2.6	22.1
15	+2	+1	−1	5.4	−0.7	16.1	6.4	−1.9	11.3
16	+2	+2	+1	5.7	−2.1	−16.2	6.7	1.9	−11.6

Table S2. Comparison of RMS three-axis angular errors under coupled attitude conditions before and after distance compensation at $L = 0$ mm .

	Pitch RMS error /(″)	Yaw RMS error /(″)	Roll RMS error /(″)
Before compensation	8.0	2.1	24.9
After compensation	5.2	2.0	17.6

Table S3. Three-axis angular errors under coupled attitude conditions before and after distance compensation at $L = -2$ mm.

Case	α / (°)	β / (°)	γ / (°)	Before compensation			After compensation		
				$\Delta\alpha$ / (″)	$\Delta\beta$ / (″)	$\Delta\gamma$ / (″)	$\Delta\alpha$ / (″)	$\Delta\beta$ / (″)	$\Delta\gamma$ / (″)
1	−2	−2	−2	17.6	10.0	40.3	−6.6	−3.8	−22.4
2	−2	−1	−1	17.5	3.3	19.5	−6.7	−4.5	21.5
3	−2	+1	+1	17.8	−6.0	−17.5	−6.4	1.2	−14.1
4	−2	+2	+2	18.2	−11.4	−37.9	−6.1	3.6	13.6
5	−1	−2	−1	18.0	10.3	18.3	−5.9	−3.8	20.5
6	−1	−1	+1	17.7	3.8	−18.7	−6.1	−4.8	−21.0
7	−1	+1	+2	18.5	−5.5	−38.6	−5.5	1.3	13.0
8	−1	+2	−2	19.0	−11.4	40.7	−4.9	3.6	−22.1
9	+1	−2	+1	2.7	11.4	−21.1	−6.4	−3.4	−15.7
10	+1	−1	+2	2.9	4.9	−41.0	−6.3	−0.8	11.9
11	+1	+1	−2	4.4	−5.5	39.2	−5.0	6.5	−22.8
12	+1	+2	−1	4.5	−10.7	18.8	−4.8	3.9	21.0
13	+2	−2	+2	1.2	12.1	−42.7	3.2	−3.2	11.3
14	+2	−1	−2	3.0	4.1	37.1	4.6	−5.2	−24.0
15	+2	+1	−1	3.2	−5.0	17.7	4.8	1.5	20.0
16	+2	+2	+1	3.4	−9.8	−19.0	4.9	4.1	−14.7

Table S4. Comparison of RMS three-axis angular errors under coupled attitude conditions before and after distance compensation at $L = -2$ mm.

	Pitch RMS error /(″)	Yaw RMS error /(″)	Roll RMS error /(″)
Before compensation	13.0	8.4	31.1
After compensation	5.6	3.8	18.6

Table S5. Three-axis angular errors under coupled attitude conditions before and after distance compensation at $L = +2$ mm.

Case	$\alpha/(°)$	$\beta/(°)$	$\gamma/(°)$	Before compensation			After compensation		
				$\Delta\alpha/('')$	$\Delta\beta/('')$	$\Delta\gamma/('')$	$\Delta\alpha/('')$	$\Delta\beta/('')$	$\Delta\gamma/('')$
1	−2	−2	−2	2.8	−5.7	24.5	−2.8	1.7	−17.5
2	−2	−1	−1	2.4	−2.6	12.9	−2.5	−2.6	15.0
3	−2	+1	+1	2.9	2.8	−14.2	−3.0	3.9	−15.3
4	−2	+2	+2	3.6	6.1	−25.8	−2.3	−1.8	11.4
5	−1	−2	−1	6.7	−5.9	12.6	6.1	1.8	15.1
6	−1	−1	+1	6.0	−2.4	−14.1	5.7	−2.4	−15.5
7	−1	+1	+2	6.8	3.1	−26.7	6.3	4.0	11.5
8	−1	+2	−2	7.3	5.8	24.9	6.7	−1.6	−17.3
9	+1	−2	+1	14.5	−5.8	−14.0	3.4	1.6	−15.1
10	+1	−1	+2	14.1	−2.8	−25.5	3.1	−2.8	11.2
11	+1	+1	−2	15.0	2.6	24.2	3.7	3.7	−17.7
12	+1	+2	−1	15.4	5.7	12.3	4.0	−1.7	15.0
13	+2	−2	+2	16.4	−5.5	−26.1	6.7	1.5	11.8
14	+2	−1	−2	16.0	−2.5	24.8	6.3	−2.5	−17.4
15	+2	+1	−1	16.3	2.8	12.8	6.9	3.8	15.2
16	+2	+2	+1	16.7	6.0	−14.0	7.3	−1.9	−15.3

Table S6. Comparison of RMS three-axis angular errors under coupled attitude conditions before and after distance compensation at $L = +2$ mm.

	Pitch RMS error /('')	Yaw RMS error /('')	Roll RMS error /('')
Before compensation	11.6	4.5	20.3
After compensation	5.1	2.6	15.0
